# Exploring the levels of homocysteine and its relationship with cognitive function in children with attention deficit hyperactivity disorder

**DOI:** 10.3389/fneur.2025.1662441

**Published:** 2025-11-03

**Authors:** Huiting Wang, Ziqi Liu, Xin Wang, Jianzhao Zhang, Jian Yang

**Affiliations:** ^1^Department of Pediatric Neurology, Capital Center For Children’s Health, Capital Medical University, Beijing, China; ^2^Department of Neonatology, Beijing Chao-Yang District Maternal and Child Healthcare Hospital, Beijing, China

**Keywords:** attention deficit hyperactivity disorder, homocysteine, cognitive function, BRIEF, children

## Abstract

**Background:**

Attention deficit hyperactivity disorder (ADHD) is a neurodevelopmental disorder frequently accompanied by cognitive dysfunction. However, the precise etiology of the cognitive impairment remains unclear. Homocysteine is recognized as a risk factor that contributes to cognitive impairment.

**Objective:**

To explore the potential changes in serum homocysteine levels in children with ADHD and to evaluate its relationship with cognitive function.

**Methods:**

In this cross-sectional and case–control study, 39 children diagnosed with ADHD were recruited from the outpatient clinic of the Capital Center For Children’s Health, Capital Medical University, along with 40 age- and sex-matched healthy children from the Health Care Department. Serum homocysteine levels were measured via the enzyme cycle method. Age and sex were incorporated into stratified analyses. Cognitive function in patients with ADHD was evaluated using the Behavior Rating Inventory of Executive Function (BRIEF).

**Results:**

Compared with the healthy individuals, patients with ADHD exhibited significantly higher serum total homocysteine levels (7.20 ± 1.19 μmol/L vs. 6.35 ± 1.11 μmol/L, *p* = 0.002). This association was prominent in younger patients (7.21 ± 1.39 μmol/L vs. 5.84 ± 0.18 μmol/L, *p* = 0.001) and male patients (7.21 ± 1.15 μmol/L vs. 6.44 ± 1.18 μmol/L, *p* = 0.010). No significant correlation was observed between serum total homocysteine levels and BRIEF scores (*p* > 0.05).

**Conclusion:**

The study indicates that compared to healthy individuals, patients with ADHD exhibit relatively high homocysteine levels, especially in younger and male patients. However, this study did not support a significant correlation between homocysteine levels and cognitive function in children with ADHD.

## 1 Introduction

Attention deficit hyperactivity disorder (ADHD) is a neurodevelopmental disorder characterized by inappropriate inattention, hyperactivity, and impulsivity that are excessive relative to the individual’s age (1). Prevalence of ADHD among the children and adolescent population worldwide is approximately 5.5% (2). However, the incidence of ADHD varies by geographic region, sex, age, and comorbidities, with lower rates reported in some countries, including Slovenia (approx. 1%) (3, 4). Individuals with ADHD typically exhibit deficits in cognitive function performance, such as working memory, response inhibition, and planning (5). These types of cognitive dysfunctions often persist from childhood into adolescence, where they have a lasting effect on the patient’s academic and daily life (6). Furthermore, comorbid conditions, such as anxiety and sleep problems, are common and may further exacerbate cognitive impairments (7, 8). Pharmacotherapy, particularly the use of psychostimulants, is an effective treatment for ADHD core symptoms and cognitive dysfunction (1, 9). However, long-term use may be associated with potential risks to growth and an increase in the incidence of cardiovascular events; thus, many patients are untreated with stimulants (1, 9, 10). Nonpharmacological interventions such as cognitive behavioral therapies have also been shown to improve cognitive function (11). However, these therapies are often characterized by a slow onset of action and have limited efficacy in addressing the core symptoms of ADHD (11). Given the limitations of both pharmacological and nonpharmacological strategies, it is imperative to not only optimize current strategies but also to identify and modify risk factors for cognitive dysfunction in ADHD. Currently, the etiology of attention deficit-related cognitive dysfunction remains uncertain, and both genetic and environmental factors contribute to its development (12, 13). In recent years, homocysteine has been implicated as a risk factor for age-related cognitive decline (14–16). For example, a meta-analysis of Alzheimer’s disease reported that each 5 μmol/L increase in the plasma homocysteine concentration elevates disease risk by 12% (15). Even among healthy geriatric cohorts, elevated homocysteine levels are associated with an accelerated rate of cognitive decline (16). In the context of ADHD, an animal study revealed that neonatal rats with hyperhomocysteinemia exhibited hyperactivity and short-term memory deficits, along with morphological changes in dendritic structure, which suggests that this model is promising for further investigations of ADHD (17). Despite these findings, the role of homocysteine in cognitive deficits associated with ADHD remains underexplored.

Several mechanisms may underlie the potential link between homocysteine levels and ADHD. First, homocysteine participates in the methionine cycle and contributes to the generation of S-adenosylmethionine, a key methyl donor for processes such as DNA modification and dopamine synthesis (18). Altered DNA methylation and dopamine transporter availability have been implicated in ADHD treatment response and pathophysiology (19). Second, homocysteine can promote inflammatory responses by upregulating proinflammatory cytokines such as interleukin-1 beta, tumor necrosis factor-alpha, and interleukin-6 (20, 21). These inflammatory mediators have also been associated with increased ADHD susceptibility (22). Third, homocysteine affects various enzymes that participate in redox reactions (23). Clinical studies have shown that compared with controls, individuals with ADHD exhibit elevated total oxidation status and oxidative stress (24). In addition, homocysteine levels are influenced by nutrition-related factors such as vitamin B6, B12, and folate levels, as well as demographic variables such as age and sex (18). Thus, factors that affect homocysteine metabolism may indirectly contribute to ADHD pathogenesis.

The few studies that have aimed to directly measure homocysteine levels in individuals with ADHD have provided inconsistent findings (25–28). For instance, Lukovac et al. reported significantly elevated homocysteine levels in children with ADHD, which correlated with symptom severity (25). Similarly, Miniksar et al. (27) reported increased ADHD prevalence with increasing homocysteine levels. In contrast, one study reported significantly lower homocysteine levels in patients with ADHD (28). These conflicting findings imply a complex, potentially modulatory role for homocysteine in ADHD. Furthermore, the association between homocysteine and cognitive dysfunction in individuals with ADHD remains poorly understood. To our knowledge, only a few studies have examined homocysteine levels in relation to Wechsler Intelligence Scale scores, with no studies have reported significant correlations (25, 28). On the basis of available evidence, our study aimed to measure and compare serum total homocysteine levels in children with ADHD (aged 6–18 years) against those in healthy individuals, with comparisons stratified by age and sex to account for the potential influence of these demographic factors. Furthermore, we explored the relationship between serum homocysteine levels and scales of cognitive function in patients with ADHD.

## 2 Methods

### 2.1 Participants

Children with ADHD were recruited from the outpatients of the Neurology Department of Capital Center For Children’s Health, Capital Medical University, as the ADHD group. All patients met the Diagnostic and Statistical Manual of Mental Disorders, fifth edition criteria for ADHD, which were diagnosed by experienced pediatricians through in-depth clinical interviews. The other inclusion criteria included age of 6–18 years, willingness of the legal guardians, and the ability to complete the Behavior Rating Inventory of Executive Function (BRIEF) questionnaire. The exclusion criteria included intellectual disability, the presence of other mental disorders, endocrine disorders, gastrointestinal disease, metabolic disease, the use of vitamin supplements or any medications, within 2 months, that could have an impact on serum total homocysteine levels, and the previous use of any ADHD medication or other nonmedicinal treatments. The individuals in the healthy control group were recruited from the Health Care Department of Capital Center For Children’s Health, Capital Medical University, for routine examinations. They were matched by age and sex to patients with ADHD. All healthy participants were confirmed to have no history of neurodevelopmental, psychiatric, or chronic medical conditions. The exclusion criteria for healthy individuals included intellectual disability, mental disorders, endocrine disorders, gastrointestinal disease, metabolic disease, use of vitamin supplements, and the use of any medications within 2 months, which could have an impact on serum total homocysteine levels.

Sample size estimation was conducted with G*Power version 3.1.9.7. For comparison of homocysteine levels between the ADHD and healthy control group, given the limited and inconsistent preliminary evidence, a medium effect size (Cohen’s d = 0.5) was assumed. With a two-sided *α* of 0.05 and power of (1-β) at 0.8, 128 participants (64 per group) were needed. To conduct correlation analysis, an assumed correlation coefficient (r) of 0.4 was used, yielding a minimum requirement of 46 participants under the same error probabilities. To ensure robust statistical power for both objectives, the final target sample size was set to 128 participants, with 64 participants per group.

### 2.2 Assessment of cognitive function

The cognitive function in ADHD patients was evaluated using the parental Version of the BRIEF questionnaire, a well-validated instrument specifically designed to evaluate executive function (29). Executive function impairment is a core deficit in the cognitive dysfunction of ADHD (13). The BRIEF is an extensive questionnaire containing eighty-six items, which are summarized into two comprehensive indices: the Behavioral Regulation Index and the Metacognition Index (30). The Behavioral Regulation Index comprises subscales including Inhibition, Shifting, and Emotional Control, while the Metacognition Index comprises the Initiate, Working Memory, Plan/Organize, Organization of Materials, and Monitoring subscales (30). These two indices together form the Global Executive Composite (31). Parents assign scores ranging from 1 to 3 to indicate their child’s typical behavioral performance. The higher total score indicates a greater severity of executive function impairment (31).

### 2.3 Measurement of homocysteine levels

Venous blood samples were obtained from participants after a 12-h overnight fast. After collection, samples were promptly transported to the clinical laboratory within 1 h. Serum was separated by centrifugation at 3000 rpm for 15 min. The enzymatic cycling method was employed for the quantification of homocysteine levels, as it represents a well-established method recommended for its high analytical specificity and suitability for clinical research (32). To maintain measurement accuracy, the detector underwent daily quality control procedures.

### 2.4 Statistical analysis

Statistical analyses were performed with SPSS version 26.0 and R version 4.5.1. Participants with less than 5% missing data were excluded from the final analysis. Continuous variable data are presented as means and standard deviations. Between-group comparisons of serum total homocysteine levels and age between children with ADHD and healthy controls were made using the Student’s t-test or the Mann–Whitney U test. Beyond the primary group comparisons, we conducted subgroup analyses to explore the potential influence of age and sex. Patients with ADHD were stratified by the median age into younger and older subgroups, and by sex into male and female subgroups. Within each subgroup, homocysteine levels were compared between the ADHD and control groups using the same statistical tests employed in the primary analysis. The chi-square test was used to evaluate differences in sex composition across the groups. Linear correlation analysis was used to conduct correlation analyses. If linear correlation analysis indicates a significant association, a regression analysis will subsequently be performed with homocysteine level as a predictor. *Post hoc* power analysis was conducted with G*Power version 3.1.9.7. Statistical significance was defined as a two-sided *p* < 0.05.

## 3 Results

### 3.1 Clinical demographics

On the basis of predefined inclusion and exclusion criteria, 10 patients were excluded from analysis. One patient was excluded for having a single missing item on the BRIEF questionnaire. 79 subjects were ultimately included, and the characteristics are shown in Tables 1, 2. The ADHD group comprised 39 children, with an average age of 8.07 ± 1.17 years. The healthy control group included 40 children, averaging 8.13 ± 1.42 years of age. In the ADHD group, 82.05% (*n* = 32) were males, while 17.95% (*n* = 7) were females. In the healthy control group, 77.50% (*n* = 31) were males, and 22.50% (*n* = 9) were females. The two groups showed no significant differences in age (*p* = 0.848) and sex distribution (*p* = 0.615).

**Table 1:** Clinical demographics of the ADHD group and the healthy control group across age groups.

Age groups	Age (mean±SD, years)		*p*-value	Sex (male/female)		*p*-value
	ADHD	Controls		ADHD	Controls	
6–8	7.10 ± 0.59	6.95 ± 0.63	0.499	16/4	17/3	1.000
8–10	9.09 ± 0.62	9.30 ± 0.90	0.584	16/3	14/6	0.451
All	8.07 ± 1.17	8.13 ± 1.42	0.848	32/7	31/9	0.615

**Table 2:** Clinical demographics of the ADHD group and the healthy control group across sex groups.

Sex	ADHD		Controls		*p*-value
	N	Age (years)	N	Age (years)	
Male	32	8.16 ± 1.20	31	8.08 ± 1.45	0.680
Female	7	7.67 ± 1.02	9	8.28 ± 1.37	0.344

### 3.2 Serum homocysteine levels

**Figure 1:**
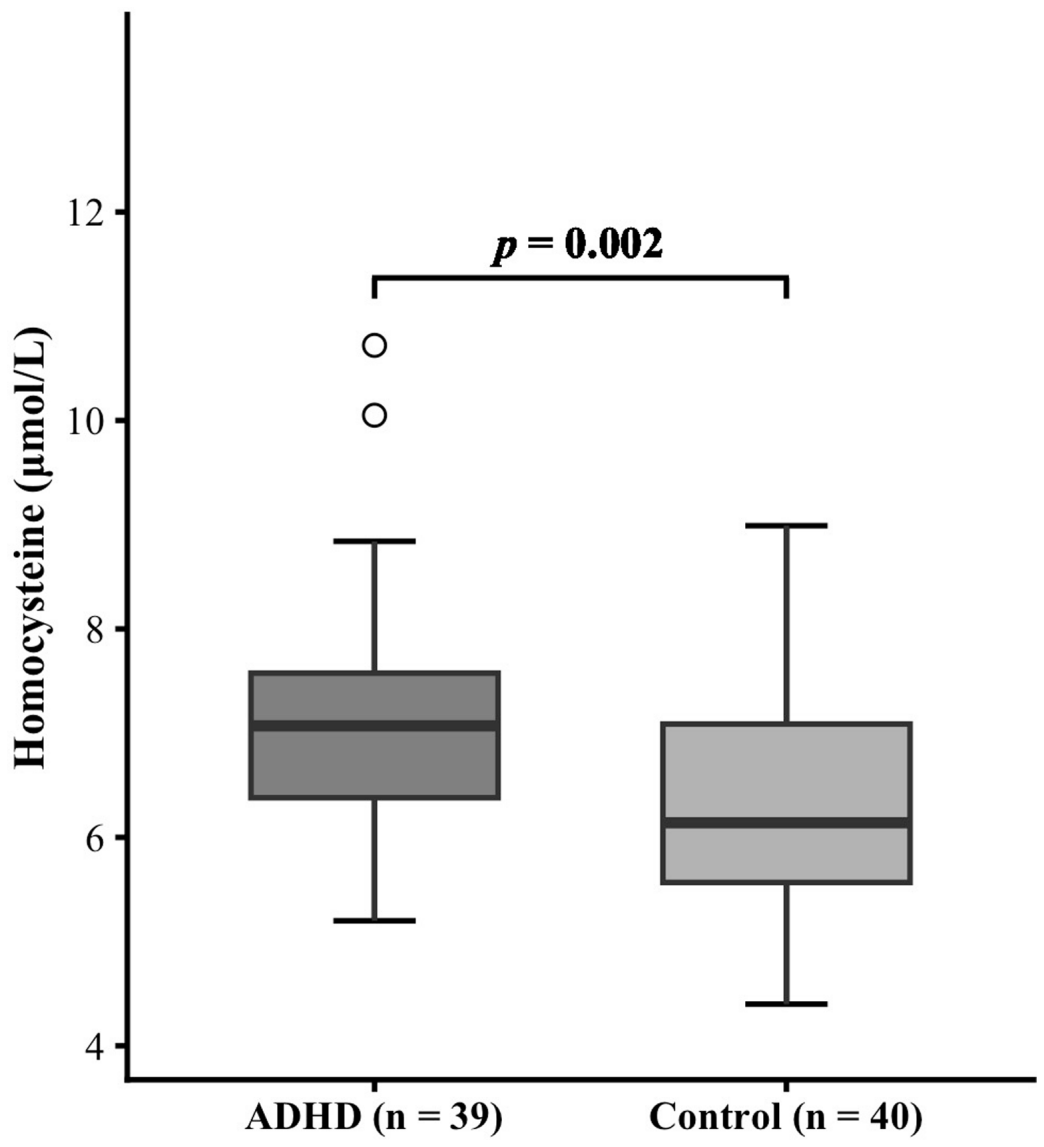
Serum homocysteine levels in the ADHD group and the healthy control group.

The ADHD group demonstrated significantly higher serum total homocysteine levels compared to the healthy control group (7.20 ± 1.19 μmol/L vs. 6.35 ± 1.11 μmol/L, *p* = 0.002). A *post hoc* power analysis confirmed the robustness of the finding, with a high statistical power of 0.90. The comparison results between the two groups are shown in Figure 1.

### 3.3 Effect of age on serum homocysteine levels

**Figure 2:**
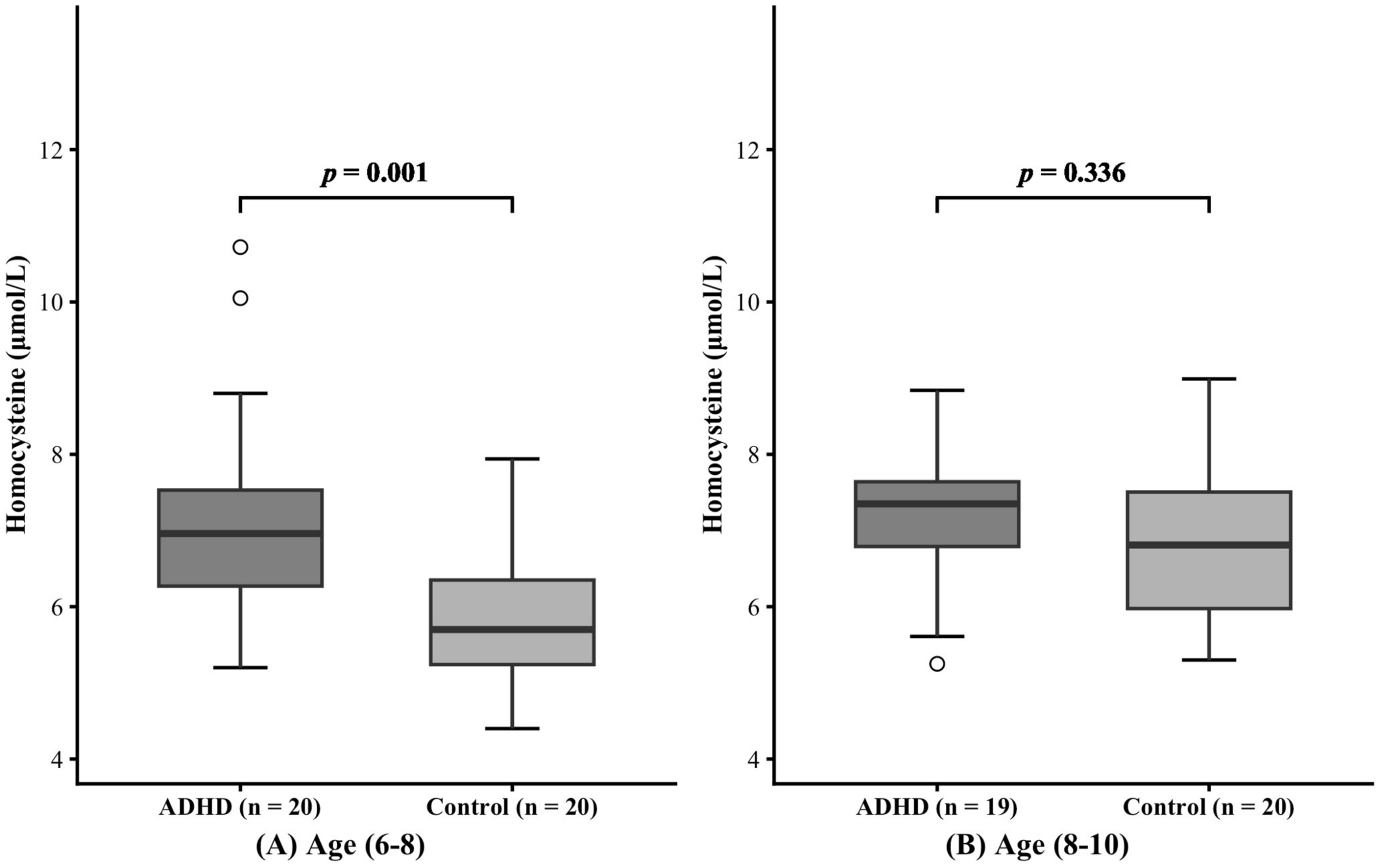
Effect of age on serum homocysteine levels. Panels **(A)** and **(B)** represent the 6-8 years and 8-10 years age groups, respectively.

During the recruitment phase, we were unable to recruit a sufficient number of adolescents aged 11 to 18. Therefore, the final analytical sample was restricted to children aged 6 to 10 years. Participants were stratified into a younger group (age 6–8 years) and an older group (age 8–10 years) according to median age. As indicated in Table 1, no significant differences in age or sex distribution were observed between the ADHD group and the healthy control group within the 6–8-year subgroup (age: *p* = 0.499; sex: *p* = 1.000) or the 8–10-year subgroup (age: *p* = 0.584; sex: *p* = 0.451). In the 6–8-year subgroup, serum homocysteine levels were higher in the ADHD group than in the healthy control group (7.21 ± 1.39 μmol/L vs. 5.84 ± 0.18 μmol/L, *p* = 0.001). However, no statistically significant difference in homocysteine levels was found between the 8–10-year subgroups (7.18 ± 0.96 μmol/L vs. 6.86 ± 1.08 μmol/L, *p* = 0.336). The effect of age on serum homocysteine levels was shown in Figure 2.

### 3.4 Effects of sex on serum homocysteine levels

**Figure 3:**
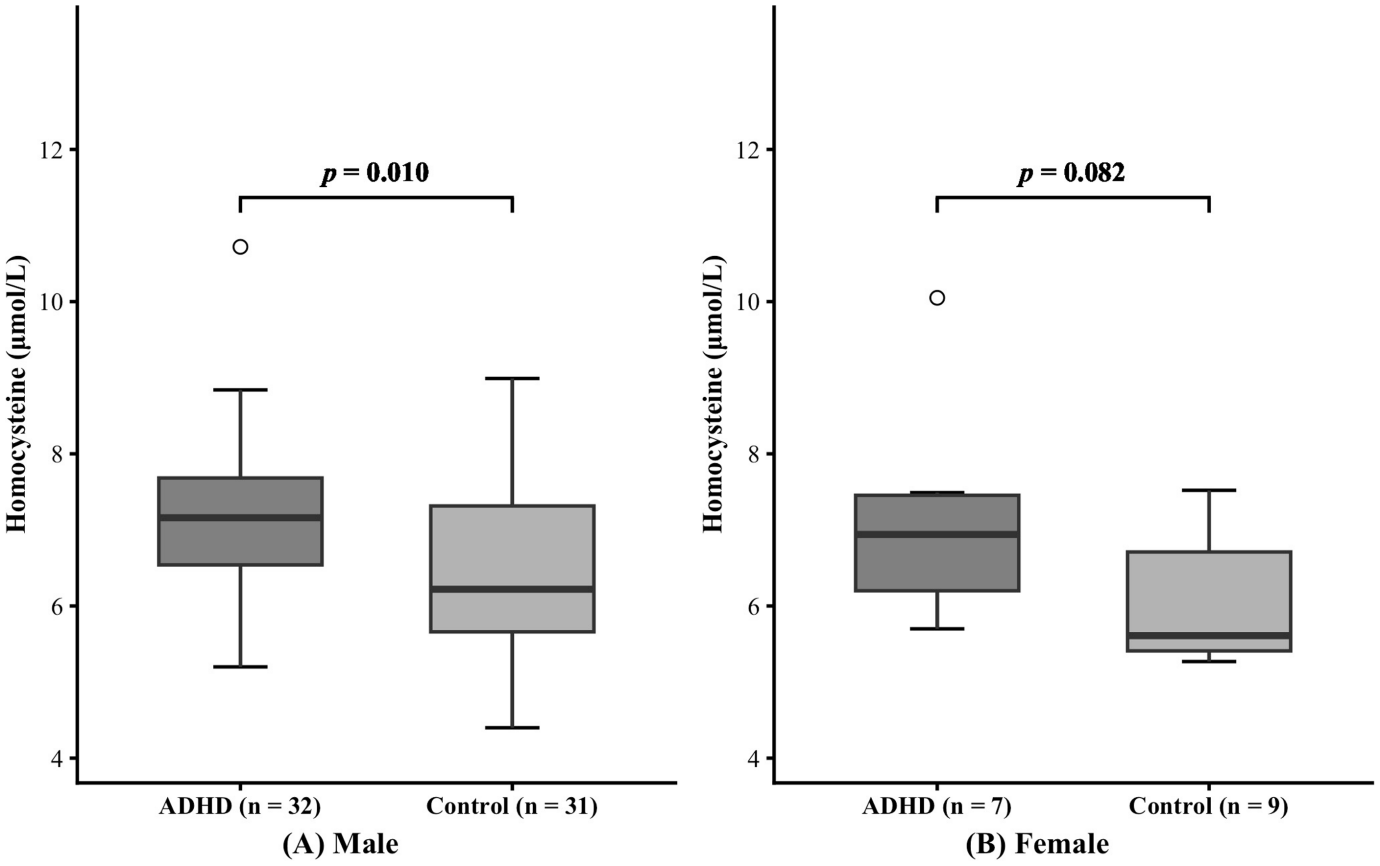
Effect of sex on serum homocysteine levels. Panels **(A)** and **(B)** represent the male group and female group, respectively.

The subjects were also classified by sex, as detailed in Table 2. Within both male and female subgroups, no significant difference in age was found (Male: *p* = 0.680; Female: *p* = 0.334). In the male subgroup, serum homocysteine levels were higher in patients with ADHD than in healthy individuals (7.21 ± 1.15 μmol/L vs. 6.44 ± 1.18 μmol/L, *p* = 0.010). Nevertheless, homocysteine levels did not differ significantly between the female subgroups (7.14 ± 1.45 μmol/L vs. 6.06 ± 0.85 μmol/L, *p* = 0.082). The comparison results of the two groups are shown in Figure 3.

### 3.5 Relationships between homocysteine levels and cognitive function

**Figure 4:**
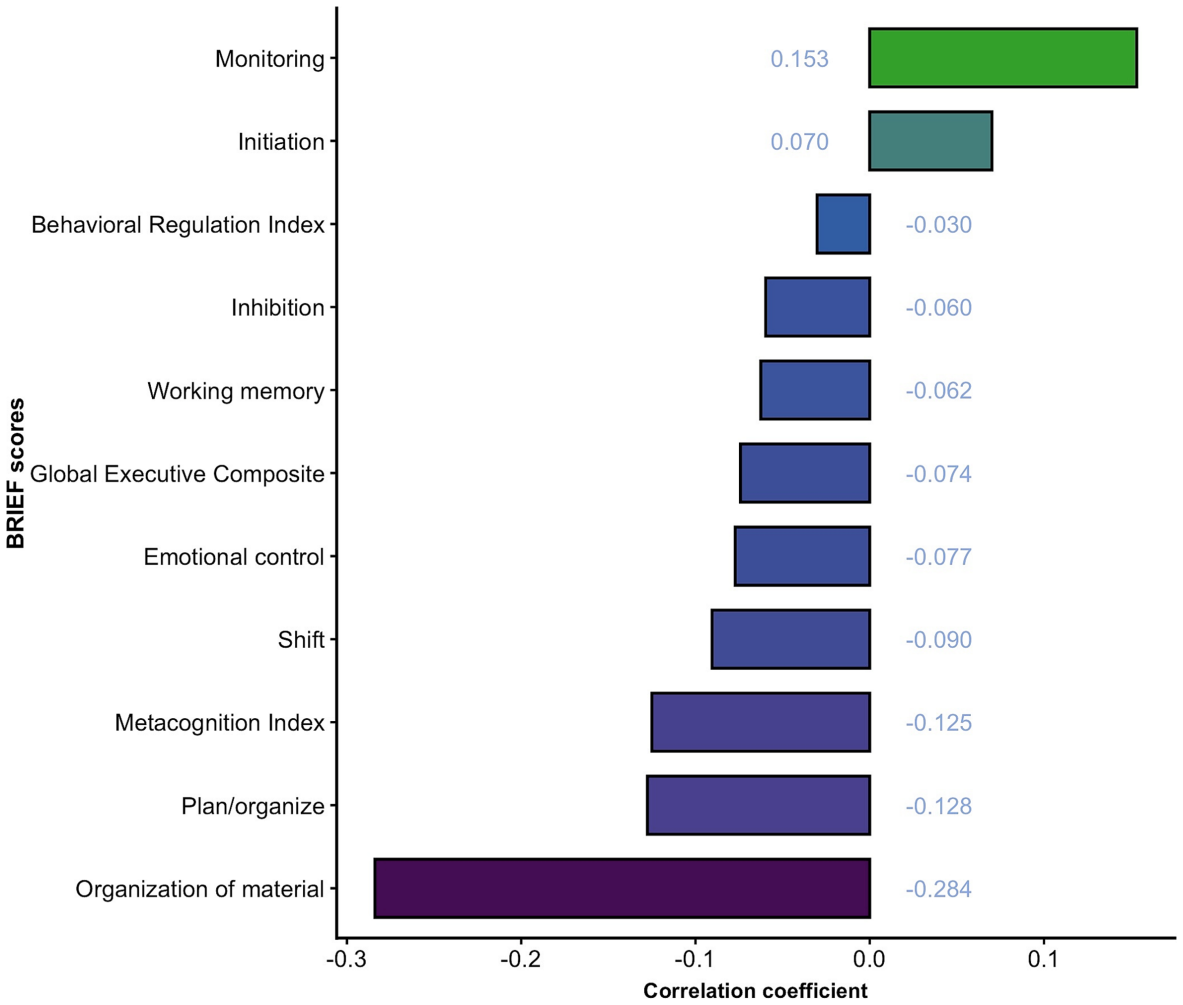
Correlations of homocysteine levels with BRIEF scores.

Exploratory linear correlation analyses across all eight executive function dimensions and three composite indices’ scores revealed no significant associations with serum total homocysteine levels in patients with ADHD (all *p*-values > 0.05), which indicates the absence of statistically significant correlations. Details are shown in Figure 4. The *post hoc* power analysis indicated less than 44% power at the observed effect size. Regression analysis was not performed based on the results of linear correlation analyses.

## 4 Discussion

Our study revealed that children with ADHD exhibit higher serum total homocysteine levels compared to healthy children. This association was particularly notable in males and in the younger age group. Our study investigated the correlation between serum homocysteine levels and BRIEF scores in children with ADHD. The results revealed no significant correlation between serum homocysteine levels and cognitive function (all *p*-values > 0.05).

### 4.1 Serum homocysteine levels in children with ADHD

Only a few studies have explored homocysteine levels in patients with ADHD, their findings lack consistency. Our results lend support to the perspective that patients with ADHD exhibit comparatively elevated serum total homocysteine levels. Miniksar et al. (27) reported that elevated serum total homocysteine levels might constitute a potential risk factor in ADHD etiology. Their research revealed that when serum total homocysteine levels exceeded 9.445umol/L, it could be a robust predictor of ADHD, as favorable and specific characteristics were observed. This is further supported by rodent models, in which neonatal homocysteine administration has been used to simulate ADHD-like phenotypes (17). However, some research contradicts the outcomes of our study and indicates that homocysteine levels are lower in both adults and children with ADHD (28, 33). It is hypothesized that in children with ADHD, lower serum levels of homocysteine might increase oxidative stress by reducing the levels of glutathione (28). However, the sizes of all the aforementioned studies were relatively limited. The possible explanations for the discrepant research findings could potentially be attributed to the relatively small sample size and the participants’ heterogeneity.

This study revealed distinct age- and sex-specific differences in homocysteine levels among children with ADHD. Increased homocysteine levels were primarily observed in younger age and male patients with ADHD, whereas no significant differences were detected in older patients and or in female patients. As a key intermediate in the methionine cycle, homocysteine provides essential methyl donors for DNA methylation and the synthesis of neurotransmitters in the body. Furthermore, homocysteine can influence brain structure and function through multiple pathways, such as by triggering inflammatory responses, inducing oxidative stress, and disrupting the electron transport chain (20, 21, 23). Early childhood represents a critical period for neurodevelopment, particularly in terms of neuronal plasticity, as young children are more susceptible to external influences (34). With respect to sex differences, sex hormones, especially estrogen, have been shown to modulate homocysteine metabolism by improving renal function to promote homocysteine conversion and clearance (35). Estrogen may also protect against homocysteine-induced inflammatory damage (36). These mechanisms may explain why no significant changes in homocysteine levels were detected in the female subjects in this study.

### 4.2 Correlation between homocysteine levels and cognitive function

Consistent with those of prior research, our study lacks a significant correlation between homocysteine levels and cognitive function in children with ADHD (25, 28). However, our results should be interpreted cautiously, as a post-hoc power analysis indicated insufficient statistical power to detect moderate effects. Previous studies have revealed that homocysteine levels are related to the cognitive function underlying cognitive impairment, dementia, and depression (14, 37). Even among healthy elderly individuals, increased serum homocysteine levels could affect memory, psychomotor speed, and global cognitive function (16). Our study does not support the conclusions of previous studies in that it failed to detect a correlation between cognitive function and serum total homocysteine levels. Notably, prior studies focused on older populations, and few studies have surveyed children. Compared with younger individuals, those who are older typically exhibit higher levels of homocysteine, and the effect of homocysteine may be amplified with age (38). Previous studies have also shown that cognitive function impairment is likely to occur when plasma homocysteine levels exceed 11 μmol/L (39). In our study, we found that patients with ADHD exhibited higher serum total homocysteine levels than healthy individuals. However, these levels still fell within the normal physiological range and did not attain the threshold indicative of hyperhomocysteinemia. It can be inferred that the serum total homocysteine levels in patients with ADHD were not high enough to cause cognitive dysfunction. In addition, the effect of homocysteine on specific brain regions or specific cognitive functions is not very clear (38). The executive function adopted in this study is only a part of the cognitive function and may not be affected by homocysteine levels.

### 4.3 Strengths and limitations

Our research findings must be comprehensively considered on the basis of the strengths and limitations. The strengths lie in the fact that our study included the comparative analysis of serum total homocysteine levels between children with ADHD and healthy controls, as well as the investigation into its relationship with cognitive ability—though no significant correlation was found. However, several limitations should be considered when interpreting our findings. First, the relatively small sample size limited statistical power to detect mild to moderate significant correlations and increased the risk of Type II errors. Second, the cross-sectional nature of our study prevents us from identifying the causality patterns between homocysteine levels and ADHD. Third, the assessment of cognitive function relied on the BRIEF questionnaire, the potential for subjective reporting bias cannot be excluded. Furthermore, the effects of other potential confounders, such as physical activity and specific nutrient levels (e.g., folate and vitamin B12), were not evaluated, which may have resulted in residual confounding. Thus, more objective cognitive measures, comprehensive baseline data, and large-scale cohorts are needed to clarify the role of homocysteine in ADHD.

## 5 Conclusion

In conclusion, this study demonstrated that serum total homocysteine levels are elevated in children with ADHD compared to healthy children, especially in younger and male patients, which implies that serum total homocysteine could potentially be a risk factor for the etiology of ADHD. However, this study did not reveal a significant correlation between homocysteine levels and cognitive function in children with ADHD. Further longitudinal studies are needed to delineate the precise role of homocysteine in ADHD development.

## Data Availability

The raw data supporting the conclusions of this article will be made available by the authors, without undue reservation.

## References

[1] Posner J Polanczyk GV Sonuga-Barke E. Attention-deficit hyperactivity disorder. Lancet. (2020) 395:450–62. doi: 10.1016/s0140-6736(19)33004-1, PMID: 31982036 PMC7880081

[2] Erskine HE Baxter AJ Patton G Moffitt TE Patel V Whiteford HA. The global coverage of prevalence data for mental disorders in children and adolescents. Epidemiol Psychiatr Sci. (2017) 26:395–402. doi: 10.1017/s2045796015001158, PMID: 26786507 PMC6998634

[3] Štuhec M Švab V Locatelli I. Prevalence and incidence of attention-deficit/hyperactivity disorder in Slovenian children and adolescents: a database study from a National Perspective. Croat Med J. (2015) 56:159–65. doi: 10.3325/cmj.2015.56.159, PMID: 25891876 PMC4410171

[4] Popit S Serod K Locatelli I Stuhec M. Prevalence of attention-deficit hyperactivity disorder (Adhd): systematic review and Meta-analysis. Europ Psychiatry. (2024) 67:e68. doi: 10.1192/j.eurpsy.2024.1786, PMID: 39381949 PMC11536208

[5] Pievsky MA McGrath RE. The neurocognitive profile of attention-deficit/hyperactivity disorder: a review of meta-analyses. Arch Clin Neuropsychol. (2018) 33:143–57. doi: 10.1093/arclin/acx055, PMID: 29106438

[6] McAuley T Crosbie J Charach A Schachar R. The persistence of cognitive deficits in remitted and unremitted Adhd: a case for the state-Independence of response inhibition. J Child Psychol Psychiatry. (2014) 55:292–300. doi: 10.1111/jcpp.12160, PMID: 24261515 PMC4263232

[7] Njardvik U Wergeland GJ Riise EN Hannesdottir DK Öst LG. Psychiatric comorbidity in children and adolescents with ADHD: a systematic review and meta-analysis. Clin Psychol Rev. (2025) 118:102571. doi: 10.1016/j.cpr.2025.102571, PMID: 40245462

[8] Sciberras E DePetro A Mensah F Hiscock H. Association between sleep and working memory in children with Adhd: a cross-sectional study. Sleep Med. (2015) 16:1192–7. doi: 10.1016/j.sleep.2015.06.006, PMID: 26429744

[9] Isfandnia F El Masri S Radua J Rubia K. The effects of chronic Administration of Stimulant and non-Stimulant Medications on executive functions in Adhd: a systematic review and Meta-analysis. Neurosci Biobehav Rev. (2024) 162:105703. doi: 10.1016/j.neubiorev.2024.105703, PMID: 38718988

[10] Stuhec M Locatelli I. Age-related pharmacotherapy of attention deficit hyperactivity disorder in Slovenia in children and adolescents: a population-based study. Europ Psychiatry. (2017) 42:129–33. doi: 10.1016/j.eurpsy.2017.01.002, PMID: 28371725

[11] Sibley MH Bruton AM Zhao X Johnstone JM Mitchell J Hatsu I. Non-pharmacological interventions for attention-deficit hyperactivity disorder in children and adolescents. Lancet Child Adolesc Health. (2023) 7:415–28. doi: 10.1016/s2352-4642(22)00381-9, PMID: 36907194 PMC10370370

[12] Demontis D Walters GB Athanasiadis G Walters R Therrien K Nielsen TT. Author correction: genome-wide analyses of Adhd identify 27 risk loci, refine the genetic architecture and implicate several cognitive domains. Nat Genet. (2023) 55:730. doi: 10.1038/s41588-023-01350-w, PMID: 36859734

[13] Schachar RJ. Fifty years of executive control research in attention-deficit/hyperactivity disorder: what we have learned and still need to know. Neurosci Biobehav Rev. (2023) 155:105461. doi: 10.1016/j.neubiorev.2023.105461, PMID: 37949153

[14] Smith AD Refsum H. Homocysteine - from disease biomarker to disease prevention. J Intern Med. (2021) 290:826–54. doi: 10.1111/joim.13279 33660358

[15] Wang Q Zhao J Chang H Liu X Zhu R. Homocysteine and folic acid: risk factors for Alzheimer's disease-an updated Meta-analysis. Front Aging Neurosci. (2021) 13:665114. doi: 10.3389/fnagi.2021.665114, PMID: 34122042 PMC8188894

[16] Nelson ME Andel R Nedelska Z Martinkova J Cechova K Markova H. The association between homocysteine and memory in older adults. J Alzheimer's Dis. (2021) 81:413–26. doi: 10.3233/jad-201558, PMID: 33814443

[17] De la Torre-Iturbe S Vázquez-Roque RA De la Cruz-López F Flores G Garcés-Ramírez L. Dendritic and behavioral changes in rats Neonatally treated with homocysteine; a proposal as an animal model to study the attention deficit hyperactivity disorder. J Chem Neuroanat. (2022) 119:102057. doi: 10.1016/j.jchemneu.2021.102057, PMID: 34871732

[18] Selhub J. Homocysteine metabolism. Annu Rev Nutr. (1999) 19:217–46. doi: 10.1146/annurev.nutr.19.1.217, PMID: 10448523

[19] Wiers CE Lohoff FW Lee J Muench C Freeman C Zehra A. Methylation of the dopamine transporter gene in blood is associated with striatal dopamine transporter availability in Adhd: a preliminary study. Eur J Neurosci. (2018) 48:1884–95. doi: 10.1111/ejn.14067, PMID: 30033547 PMC6113083

[20] Dos Santos TM Ramires Júnior OV Alves VS Coutinho-Silva R Savio LEB Wyse ATS. Hyperhomocysteinemia alters cytokine gene expression, cytochrome C oxidase activity and oxidative stress in striatum and cerebellum of rodents. Life Sci. (2021) 277:119386. doi: 10.1016/j.lfs.2021.119386, PMID: 33774024

[21] Kumar M Sandhir R. Hydrogen sulfide suppresses homocysteine-induced glial activation and inflammatory response. Nitric Oxide. (2019) 90:15–28. doi: 10.1016/j.niox.2019.05.008, PMID: 31146011

[22] Allred EN Dammann O Fichorova RN Hooper SR Hunter SJ Joseph RM. Systemic inflammation during the first postnatal month and the risk of attention deficit hyperactivity disorder characteristics among 10 year-old children born extremely preterm. J Neuroimmune Pharmacol. (2017) 12:531–43. doi: 10.1007/s11481-017-9742-9, PMID: 28405874 PMC6508968

[23] Jan M Cueto R Jiang X Lu L Sardy J Xiong X. Molecular processes mediating Hyperhomocysteinemia-induced metabolic reprogramming, redox regulation and growth inhibition in endothelial cells. Redox Biol. (2021) 45:102018. doi: 10.1016/j.redox.2021.102018, PMID: 34140262 PMC8282538

[24] Corona JC. Role of oxidative stress and neuroinflammation in attention-deficit/hyperactivity disorder. Antioxidants. (2020) 9:39. doi: 10.3390/antiox9111039, PMID: 33114154 PMC7690797

[25] Lukovac T Hil OA Popović M Jovanović V Savić T Pavlović AM. Serum biomarker analysis in pediatric ADHD: implications of homocysteine, vitamin B12, vitamin D, ferritin, and iron levels. Children. (2024) 11:497. doi: 10.3390/children11040497, PMID: 38671715 PMC11048887

[26] Yektaş Ç Alpay M Tufan AE. Comparison of serum B12, folate and homocysteine concentrations in children with autism Spectrum disorder or attention deficit hyperactivity disorder and healthy controls. Neuropsychiatr Dis Treat. (2019) 15:2213–9. doi: 10.2147/ndt.S212361, PMID: 31496704 PMC6689552

[27] Miniksar DY Cansız MA Kılıç M Göçmen AY. Relationship between sleep problems and Chronotypes of children and adolescents with attention deficit and hyperactivity disorder and serum GABA, glutamate and homocysteine levels. Chronobiol. Int. (2022) 39:386–97. doi: 10.1080/07420528.2021.2018452, PMID: 34961406

[28] Altun H Şahin N Belge Kurutaş E Güngör O. Homocysteine, pyridoxine, folate and vitamin B12 levels in children with attention deficit hyperactivity disorder. Psychiatr Danub. (2018) 30:310–6. doi: 10.24869/psyd.2018.310, PMID: 30267523

[29] Qian Y Wang YF. [Reliability and Validity of Behavior Rating Scale of Executive Function Parent Form for School Age Children in China]. Beijing da xue xue bao Yi xue ban = Journal of Peking University Health sciences (2007) 39:277–83. Epub 2007/06/19. doi: 10.19723/j.issn.1671-167x.2007.03.015 17572784

[30] Baron IS. Behavior rating inventory of executive function. Child Neuropsychol. (2000) 6:235–8. doi: 10.1076/chin.6.3.235.3152 11419452

[31] McCandless S L OL. The clinical utility of the behavior rating inventory of executive function (Brief) in the diagnosis of ADHD. J Atten Disord. (2007) 10:381–9. doi: 10.1177/1087054706292115 17449837

[32] Roberts RF Roberts WL. Performance characteristics of a recombinant enzymatic cycling assay for quantification of total homocysteine in serum or plasma. Clin Chim Acta. (2004) 344:95–9. doi: 10.1016/j.cccn.2004.02.013, PMID: 15149876

[33] Karababa İF Savas SN Selek S Cicek E Cicek EI Asoglu M. Homocysteine levels and oxidative stress parameters in patients with adult Adhd. J Atten Disord. (2017) 21:487–93. doi: 10.1177/1087054714538657, PMID: 24994877

[34] Jing JQ Jia SJ Yang CJ. Physical activity promotes brain development through serotonin during early childhood. Neuroscience. (2024) 554:34–42. doi: 10.1016/j.neuroscience.2024.07.015, PMID: 39004411

[35] Niu XN Wen H Sun N Yang Y Du SH Xie R. Estradiol and Hyperhomocysteinemia are linked predominantly through part renal function indicators. Front Endocrinol. (2022) 13:817579. doi: 10.3389/fendo.2022.817579, PMID: 35663317 PMC9157416

[36] Yu T Zhang D Yang Y Jiang Y Wang J Li J. 17β estradiol activates autophagy and attenuates homocysteine mediated inflammation in endothelial cells through Pi3k Akt Mtor signaling. Sci Rep. (2025) 15:24270. doi: 10.1038/s41598-025-08797-3, PMID: 40624227 PMC12234725

[37] Zhou H Zhong X Chen B Wu Z Zhang M Mai N. Interactive effects of elevated homocysteine and late-life depression on cognitive impairment. J Affect Disord. (2020) 277:212–7. doi: 10.1016/j.jad.2020.08.022, PMID: 32829197

[38] Luzzi S Cherubini V Falsetti L Viticchi G Silvestrini M Toraldo A. Homocysteine, cognitive functions, and degenerative dementias: state of the art. Biomedicine. (2022) 10:741. doi: 10.3390/biomedicines10112741, PMID: 36359260 PMC9687733

[39] Silberstein RB Pipingas A Scholey AB. Homocysteine modulates brain functional connectivity in a memory retrieval task. J. Alzheimers Dis. (2022) 90:199–209. doi: 10.3233/jad-220612, PMID: 36093708

